# Psychosocial factors and cancer incidence (PSY‐CA): Protocol for individual participant data meta‐analyses

**DOI:** 10.1002/brb3.2340

**Published:** 2021-09-02

**Authors:** Lonneke A. van Tuijl, Adri C. Voogd, Alexander de Graeff, Adriaan W. Hoogendoorn, Adelita V. Ranchor, Kuan‐Yu Pan, Maartje Basten, Femke Lamers, Mirjam I. Geerlings, Jessica G. Abell, Philip Awadalla, Marije F. Bakker, Aartjan T. F. Beekman, Ottar Bjerkeset, Andy Boyd, Yunsong Cui, Henrike Galenkamp, Bert Garssen, Sean Hellingman, Martijn Huisman, Anke Huss, Melanie R. Keats, Almar A. L. Kok, Annemarie I. Luik, Nolwenn Noisel, N. Charlotte Onland‐Moret, Yves Payette, Brenda W. J. H. Penninx, Lützen Portengen, Ina Rissanen, Annelieke M. Roest, Judith G. M. Rosmalen, Rikje Ruiter, Robert A. Schoevers, David M. Soave, Mandy Spaan, Andrew Steptoe, Karien Stronks, Erik R. Sund, Ellen Sweeney, Alison Teyhan, Ilonca Vaartjes, Kimberly D. van der Willik, Flora E. van Leeuwen, Rutger van Petersen, W. M. Monique Verschuren, Frank Visseren, Roel Vermeulen, Joost Dekker

**Affiliations:** ^1^ Department of Internal Medicine Maasstad Hospital Rotterdam The Netherlands; ^2^ Department of Internal Medicine Division of Medical Oncology GROW Maastricht University Medical Centre Maastricht The Netherlands; ^3^ Department of Epidemiology GROW Maastricht University Maastricht The Netherlands; ^4^ Department of Research Netherlands Comprehensive Cancer Organization (IKNL) Utrecht The Netherlands; ^5^ Department of Medical Oncology Cancer Center University Medical Center University of Utrecht Utrecht The Netherlands; ^6^ Amsterdam UMC Department of Psychiatry Amsterdam Public Health Research Institute Vrije Universiteit Amsterdam The Netherlands; ^7^ GGZ inGeest Specialized Mental Health Care Amsterdam The Netherlands; ^8^ Julius Center for Health Sciences and Primary Care University Medical Center Utrecht and Utrecht University Utrecht the Netherlands; ^9^ Department of Behavioural Science and Health University College London London UK; ^10^ Ontario Institute for Cancer Research Toronto Ontario Canada; ^11^ Department of Molecular Genetics University of Toronto Toronto Ontario Canada; ^12^ Dalla Lana School of Public Health University of Toronto Toronto Ontario Canada; ^13^ Faculty of Nursing and Health Sciences Nord University Bodø Norway; ^14^ Faculty of Medicine and Health Sciences Department of Mental Health Norwegian University of Science and Technology Trondheim Norway; ^15^ Bristol Medical School, Population Health Sciences University of Bristol Bristol UK; ^16^ Atlantic Partnership for Tomorrow's Health Faculty of Health Dalhousie University Halifax Nova Scotia Canada; ^17^ Department of Public and Occupational Health Amsterdam UMC and Amsterdam Public Health Research Institute University of Amsterdam Amsterdam Netherlands; ^18^ Department of Mathematics Wilfrid Laurier University Waterloo Canada; ^19^ Amsterdam UMC Department of Epidemiology & Data Science Amsterdam Public Health institute Vrije Universiteit Amsterdam Amsterdam The Netherlands; ^20^ Department of Sociology Vrije Universiteit Amsterdam Amsterdam The Netherlands; ^21^ Institute for Risk Assessment Sciences Utrecht University Utrecht The Netherlands; ^22^ School of Health and Human Performance Faculty of Health Dalhousie University Halifax Canada; ^23^ Department of Epidemiology Erasmus MC–University Medical Center Rotterdam The Netherlands; ^24^ Department of Child and Adolescent Psychiatry/Psychology Erasmus MC–University Medical Center Rotterdam The Netherlands; ^25^ CARTaGENE, CHU Sainte‐Justine, 3175 Chemin de la Côte‐Sainte‐Catherine Montréal Québec Canada; ^26^ Department of Developmental Psychology University of Groningen Groningen The Netherlands; ^27^ Departments of Psychiatry and Internal Medicine University Medical Center Groningen University of Groningen Groningen The Netherlands; ^28^ Department of Internal Medicine, Maasstad Rotterdam The Netherlands; ^29^ Department of Psychiatry University Medical Center Groningen University of Groningen Groningen the Netherlands; ^30^ Division of Psychosocial Research and Epidemiology The Netherlands Cancer Institute Amsterdam The Netherlands; ^31^ Department of Public Health and Nursing HUNT Research Centre Faculty of Medicine and Health Sciences Norwegian University of Science and Technology (NTNU) Trondheim Norway; ^32^ Levanger hospital Nord‐Trøndelag Hospital Trust Levanger Norway; ^33^ Centre for Nutrition, Prevention and Health Services National Institute for Public Health and the Environment Utrecht the Netherlands; ^34^ Department of Vascular Medicine University Medical Center Utrecht Utrecht The Netherlands; ^35^ Amsterdam Public Health Research Institute Amsterdam Noord‐Holland The Netherlands; ^36^ Department of Rehabilitation Medicine and Department of Psychiatry Amsterdam UMC ‐ VUMC Amsterdam Noord‐Holland The Netherlands

**Keywords:** cancer risk, depression, health behaviors, meta‐analysis, psycho‐oncology

## Abstract

**Objectives:**

Psychosocial factors have been hypothesized to increase the risk of cancer. This study aims (1) to test whether psychosocial factors (depression, anxiety, recent loss events, subjective social support, relationship status, general distress, and neuroticism) are associated with the incidence of any cancer (any, breast, lung, prostate, colorectal, smoking‐related, and alcohol‐related); (2) to test the interaction between psychosocial factors and factors related to cancer risk (smoking, alcohol use, weight, physical activity, sedentary behavior, sleep, age, sex, education, hormone replacement therapy, and menopausal status) with regard to the incidence of cancer; and (3) to test the mediating role of health behaviors (smoking, alcohol use, weight, physical activity, sedentary behavior, and sleep) in the relationship between psychosocial factors and the incidence of cancer.

**Methods:**

The psychosocial factors and cancer incidence (PSY‐CA) consortium was established involving experts in the field of (psycho‐)oncology, methodology, and epidemiology. Using data collected in 18 cohorts (*N* = 617,355), a preplanned two‐stage individual participant data (IPD) meta‐analysis is proposed. Standardized analyses will be conducted on harmonized datasets for each cohort (stage 1), and meta‐analyses will be performed on the risk estimates (stage 2).

**Conclusion:**

PSY‐CA aims to elucidate the relationship between psychosocial factors and cancer risk by addressing several shortcomings of prior meta‐analyses.

## Abstract 2

Psychosocial factors and cancer incidence (PSY‐CA) consortium endeavor to understand the role of several different psychosocial factors—including depression and anxiety—in the onset of cancer. The study will employ the use of individual participant data meta‐analyses, combining results from several large, international cohorts with longitudinal designs. Furthermore, PSY‐CA aims to elucidate the role of health behaviors in the relationship between psychosocial factors and cancer risk.

## 1 INTRODUCTION

Psychosocial factors such as depression, general distress, and low social support have long been theorized to increase cancer risk (Dalton et al., 2002). Findings from prior research studying the association between psychosocial factors and cancer are mixed. Two meta‐analyses focusing on depression concluded that there was a small, potentially trivial, effect on cancer risk (McGee et al., 1994; Oerlemans et al., 2007). Another meta‐analysis of the published literature indicated that depression (combined hazards ratio [HR] = 1.29), psychosocial factors relating to stress‐prone personality or poor coping style (combined HR = 1.08), and psychosocial factors relating to emotional distress or poor quality of life (combined HR = 1.13) increased the risk for all cancer outcomes and, when collapsing psychosocial factors across subtypes, especially for lung cancer (combined HR = 1.23) (Chida et al., 2008). However, the included studies varied greatly in the psychosocial factors investigated and the cancer endpoint of interest. It is crucial to use clearly and specifically defined psychosocial factors as they can lead to distinct physiological and behavioral effects (O'Donovan et al., 2010). These effects may increase risk for specific cancers given their unique etiologies. Furthermore, published studies vary greatly in the confounders adjusted for (if any), making reliability and interpretation of outcome debatable. Rather than including studies where analyses have been determined by the original authors, two‐stage individual participant data (IPD) meta‐analysis refers to the (re‐)analysis of original data for each cohort using a standardized approach (stage 1) before combining in a meta‐analysis (stage 2) (Tierney et al., 2020). IPD meta‐analyses of cohorts have the potential to produce more reliable results than meta‐analyses of published findings (Stewart & Parmar, 1993) as one can ensure a consistent definition of the psychosocial factors, specific cancer endpoints, and key confounders adjusted for across all included cohorts.

Evidence remains limited regarding how psychosocial factors increase cancer risk. Theory postulates several possible, potentially interrelated, pathways that link psychosocial factors and cancer, including angiogenesis, endocrine mechanisms, immunosuppression, impairments in DNA repair, and inflammation (Lutgendorf et al., 2007). While health behaviors as a potential pathway between psychosocial factors and cancer have received little attention, they deserve consideration given the established relationship between health behaviors and psychosocial factors (Strine et al., 2008; Verger et al., 2009), and between health behaviors and cancer (Biswas et al., 2015; Chen et al., 2018; Kerr et al., 2017). To date, studies have most often considered health behaviors as confounders, rather than playing a direct role in the association between psychosocial factors and cancer risk. If health behaviors explain the relationship between psychosocial factors and cancer risk, this may justify offering health behavior interventions in at‐risk groups such as individuals who are depressed and smoke.

The effects of psychosocial factors on cancer development may depend on the presence or absence of health behaviors, somatic factors or demographic factors. If both psychosocial factors and health behaviors or somatic factors play a causal role in cancer development, they may interact with each other. If there is interaction, the presence of both factors puts a person at a higher risk for cancer than what would be expected based on the sum of the risk of each factor alone. This may be the case when psychosocial factors and health behaviors or somatic factors affect cancer development via the same or interrelated pathways. Factors which have been related to cancer risk and may interact with psychosocial factors include smoking (Knekt et al., 1998), weight (Kerr et al., 2017), alcohol use (Pelucchi et al., 2011), physical activity (Kerr et al., 2017), sedentary behavior (Kerr et al., 2017), sleep duration and sleep quality (Hurley et al., 2015), menopausal status (Trichopoulos et al., 1972), hormone replacement therapy (Vecchia et al., 2001), age (Thakkar et al., 2014), sex (White et al., 2018), and education level (Mouw et al., 2008). For example, in one study, depressive symptoms increased the risk of colorectal cancer particularly in overweight women (Kroenke et al., 2005), and another study found that the effect of depressive symptoms on cancer risk was increased at higher levels of cigarette smoking (Linkins & Comstock, 1990). Studying interactions provides insight into the mechanisms leading to cancer development and also shows for which subgroups the association between psychosocial factors and cancer incidence is most prominent and thus could benefit most from preventive interventions.

Health behaviors may not only interact with psychosocial factors, but may also function as mediators situated in the pathway from psychosocial factors to the development of cancer. Symptoms of depression, for example, have been linked to smoking initiation and the amount of smoking (Steuber & Danner, 2006), increased alcohol use (Bulloch et al., 2012), weight gain, weight loss, obesity (Blaine, 2008), decreased physical activity (Roshanaei‐Moghaddam et al., 2009), increased sedentary behavior (Roshanaei‐Moghaddam et al., 2009), and sleep disturbances (Benca et al., 1997), all of which have subsequently been associated with an increased cancer risk (Biswas et al., 2015; Chen et al., 2018; Kerr et al., 2017). While weight is not a health behavior, we refer to this as a health behavior given the association with several other health behaviors, specifically diet and physical activity. Despite numerous allusions to the potential mediating role of health behaviors in the relationship between psychosocial factors and cancer (Chida et al., 2008; Dalton et al., 2002), there are remarkably few studies in which this has been tested.

**FIGURE 1:**
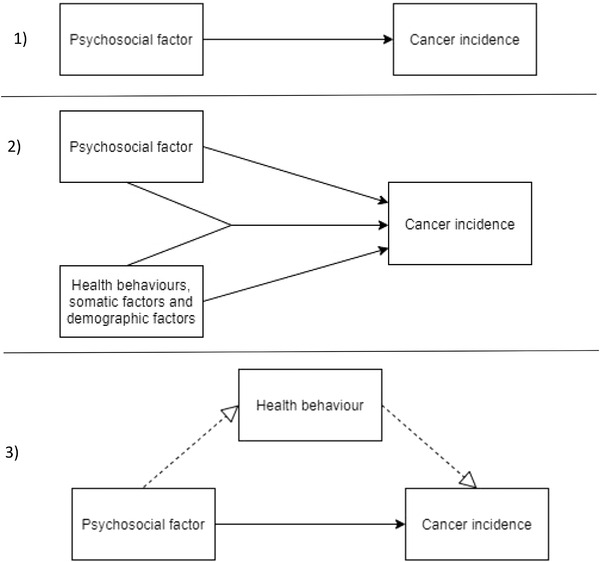
The three models that are researched in psychosocial factors and cancer incidence (PSY‐CA): (1) the longitudinal association between psychosocial factors and cancer incidence, (2) the interaction between psychosocial factors and health behaviors, somatic factors, and demographic factors in cancer incidence, and (3) the mediating role of health behaviors in the longitudinal association between psychosocial factors and cancer incidence

The psychosocial factors and cancer incidence (PSY‐CA) consortium was established to investigate whether a variety of psychosocial factors increase the risk of cancer. The investigated psychosocial factors include diagnosed depressive disorder and depressive symptoms (Jia et al., 2017) (here forth referred to as depression), diagnosed anxiety disorder and anxiety symptoms (Chen et al., 2018) (here forth referred to as anxiety), (recent) loss events (Dalton et al., 2002), perceived low social support (Idahl et al., 2018), relationship status (Randi et al., 2004), general distress (Peled et al., 2008), and neuroticism (Schapiro et al., 2001). The cancer endpoints include any cancer and the four most prevalent cancers worldwide (excluding nonmelanoma skin cancer): breast cancer, lung cancer, prostate cancer, and colorectal cancer. We also categorize cancers for which common causal factors are known, namely: smoking‐related cancers and alcohol‐related cancers, as psychosocial factors may increase smoking and alcohol use (Strine et al., 2008; Verger et al., 2009), thereby increasing risk for these cancers. The goals of the PSY‐CA consortium are (1) to test whether psychosocial factors (depression, anxiety, recent loss events, subjective social support, relationship status, general distress, and neuroticism) are associated with the incidence of any cancer (breast, lung, prostate, colorectal, smoking‐related cancer, and alcohol‐related cancer); (2) to test the interaction between psychosocial factors and factors related to cancer risk (smoking, alcohol use, weight, physical activity, sedentary behavior, sleep, age, sex, education, hormone replacement therapy, and menopausal status) in the incidence of cancer; (3) to test the mediating role of health behaviors (smoking, alcohol use, weight, physical activity, sedentary behavior, and sleep) in the relationship between psychosocial factors and the incidence of cancer (see Figure 1). Specific hypotheses have been formulated (Appendix 1).

## 2 METHODS

### 2.1 Design overview

Preplanned two‐stage IPD meta‐analyses are performed. We apply the Maelstrom guidelines (Fortier et al., 2016) to create harmonized variables across the 18 cohorts. Data are analyzed in each cohort (stage 1) and the outputs are used in a meta‐analysis (stage 2).

### 2.2 PSY‐CA consortium

The consortium consists of the steering group (LvT, JD, AV, AdG, and AVR), three main researchers (LvT, MB, and K‐YP), representatives from each participating cohort, and selected experts in the field of psycho‐oncology, epidemiology, methodology, and statistics. Meetings are held at least two times a year with the first formal consortium meeting having taken place on March 2019. During the meetings, consensus is reached on the objectives, approach, and interpretation of findings. The project leader (JD), the steering group, and the representatives of the Dutch cohorts are responsible for the formal management of the study.

### 2.3 Preregistration

The PSY‐CA study has been preregistered in PROSPERO:

https://www.crd.york.ac.uk/prospero/display_record.php?ID = CRD42020157677 (study aim 1), https://www.crd.york.ac.uk/prospero/display_record.php?ID = CRD42020181623 (study aim 2), and https://www.crd.york.ac.uk/prospero/display_record.php?ID = CRD42020193716 (study aim 3).

### 2.4 Ethics

The ethics approval for PSY‐CA was waived by the Medical Ethics Review Committee of VU University Medical Center (2018.101). For inclusion in PSY‐CA, ethics approval was granted for each study by the local institution or through appropriate national research governance frameworks.

### 2.5 Inclusion/exclusion criteria

#### 2.5.1 Cohorts

Cohorts were eligible to take part in PSY‐CA if following criteria were met:

1. a valid and reliable measure of depression, anxiety, recent loss events, social support, general distress, and/or neuroticism;
2. availability of an objective measure of cancer diagnosis during follow‐up or the potential to get this information through, for example, linkage with a cancer registry;
3. availability of data regarding smoking, alcohol, sex, and age; and
4. a prospective study design (i.e., psychosocial factors were measured before cancer incidence).

Cohorts were not eligible if there was no information about a history of cancer at baseline. Initially, relatively objective social support (i.e., social network size) and hopelessness were concepts also included in the first criterion, however as most cohorts did not have a measure of this, these concepts were subsequently dropped. Objective social support was replaced with relationship status. One cohort—Prospect‐EPIC (see Table 1)—initially appeared to have information about depression and anxiety diagnoses through a psychiatric registry. However, on closer inspection, this data appeared to be incomplete. As relationship status was measured in Prospect‐EPIC, it remained included in the study.

**TABLE 1:** Overview cohorts participating in the psychosocial factors and cancer incidence (PSY‐CA) study

Name of Cohort	Number of subcohorts included^b^	Organizations (country)	Number of participants^a^	Approx. max follow‐up duration (years) for cancer diagnosis	Reference
Ontario Health Study	1	University of Toronto (Canada)	163,257	10	(Borugian et al., 2010; Dummer et al., 2018)
Lifelines	1	University Medical Center Groningen (The Netherlands)	152,000	13	(Scholtens et al., 2015)
Nord‐Trøndelag Health Study (HUNT)	2	Norwegian University of Science and Technology (Norway)	62,237	13–24	(Krokstad et al., 2013)
CARTaGENE	1	Centre Hospitalier Universitaire Sainte Justine (Canada)	43,000	10	(Awadalla et al., 2013; Borugian et al., 2010; Dummer et al., 2018)
Atlantic PATH	1	Dalhousie University (Canada)	34,169	10	(Borugian et al. 2010; Sweeney et al., 2017)
European Prospective Investigation into Cancer and Nutrition (MORGEN‐EPIC)	1	National Institute for Public Health and the Environment (RIVM) (The Netherlands)	23,100	24	(Beulens et al., 2010; Riboli, 1992, 2002)
Healthy Life in an Urban Setting (HELIUS)	1	Amsterdam University Medical Centers and Amsterdam Municipal Health Service (The Netherlands)	19,932	8	(Snijder et al., 2017; Stronks et al., 2013)
European Prospective Investigation into Cancer and Nutrition (Prospect‐EPIC)	1	University Medical Center Utrecht (The Netherlands)	17,357	24	(Beulens et al., 2010; Boker et al., 2001; Riboli, 1992, 2002)
Dutch occupational and Environmental Health Cohort Study (AMIGO)	1	Utrecht University (The Netherlands)	14,829	5	(Slottje et al., 2014)
Avon Longitudinal Study of Parents and Children (ALSPAC)	1	University of Bristol (England)	14,541	20	(Fraser et al., 2013)
Second Manifestations of ARTerial disease (SMART)	1	University Medical Center Utrecht (The Netherlands)	11,881	12	(Simons et al., 1999)
Rotterdam Study	3	Erasmus MC University Medical Center (The Netherlands)	11,740	8–14	(Hofman et al., 2015)
English Longitudinal Study of Ageing (ELSA)	1	University College London (England)	11,391	14	(Steptoe et al., 2013)
Whitehall‐II study (WH‐II)	1	University College London (England)	10,308	11	(Marmot & Brunner, 2005)
OMEGA‐II	1	The Netherlands Cancer Institute (The Netherlands)	10,000	8	(van den Belt‐Dusebout et al., 2016; Spaan et al., 2016)
Utrecht Health Project (UHP)	2	University Medical Center Utrecht (The Netherlands)	10,000	11–19	(Grobbee et al., 2005)
Longitudinal Aging Study Amsterdam (LASA)	1	Amsterdam University Medical Centers (The Netherlands)	4632	28	(Hoogendijk et al., 2016; Huisman et al., 2011)
Netherlands Study of Depression and Anxiety (NESDA)	1	Amsterdam University Medical Centers, on behalf of the NESDA consortium (www.nesda.nl) (The Netherlands)	2981	15	(Penninx et al., 2008)

*Note*: In some cohorts a measurement wave other than baseline is used in PSY‐CA due to the absence of a measure relating to one of the psychosocial factors outlined in the hypotheses.

^a^ This is before applying any exclusion criteria (e.g., a history of cancer) and based on baseline adult sample sizes.

^b^ Subcohorts are limited to those that are treated as subcohorts in the meta‐analyses. For certain cohorts, subcohorts were combined where subsample sizes were too small otherwise (i.e., <1000) and combining resulted in minimal or no loss of data.

#### 2.5.2 Participants

Across all analyses, participants were excluded if they had a cancer diagnosis (based on [cancer] registry data or self‐report) at baseline or in the past (including in situ carcinomas and neoplasms of undetermined behavior [i.e., benign/malignant status undetermined]), with the exception of nonmelanoma skin cancer. Participants who had refused linkage with an external registry were also excluded from any analysis. People with a cancer diagnosis within one year from baseline were excluded from the analysis.

### 2.6 Search strategy and eligible studies

In preparation of PSY‐CA, a feasibility study was conducted to identify potential cohorts (December 2015 toMarch 2017). An extensive search for all relevant Dutch cohorts was conducted using the network of experts participating in PSY‐CA. The coordinators of these cohorts were approached and invited to take part in PSY‐CA. In order to increase the number of cohorts, international cohorts that fulfilled the inclusion criteria were identified through the BioShare consortium (which is now linked to the Public Population Project in Genomics and Society) (http://www.p3gconsortium.org/about‐p3g), Integrative Analysis of Longitudinal Studies of Aging network (www.ialsa.org/). Consortium members were also asked if they knew of the existence of any international cohorts that met the inclusion criteria. In addition, a literature review was conducted in preparation of the feasibility study.

During the feasibility study, coordinators of the candidate cohorts were contacted to check whether the inclusion and exclusion criteria were met, and to outline any other potential issues related to costs or ethical issues, for example. The included cohorts (11 cohorts in the Netherlands and seven cohorts in the United Kingdom, Norway, and Canada) are outlined in Table 1.

PSY‐CA is set up in such a way that after the project has finished, additional cohorts can be incorporated by applying the harmonization manual (outlined in data handling below) to the data and running the standardized analyses scripts.

### 2.7 Variables

#### 2.7.1 Psychosocial factors

The relationship between the following seven psychosocial factors and cancer incidence is analyzed: depressive symptoms or clinical depression (i.e., major depressive disorder, dysthymia), anxiety symptoms or anxiety disorders (excluding specific phobias*)*, recent loss events (defined specifically as the loss of an immediate family member or partner in the last 12 months), perceived social support, relationship status, general distress (specified as scored on the Mental Health Inventory of the Short‐form health survey (SF‐36) or the RAND36 (Ware & Sherbourne, 1992)), and neuroticism. Only validated or previously published measures of the psychosocial factors are used.

#### 2.7.2 Cancer

The primary outcomes in PSY‐CA are incidence of all cancers, smoking‐ and alcohol‐related cancers, and the four most prevalent cancers in the Netherlands: breast cancer (females only), colorectal cancer, lung cancer, and prostate cancer (males only). Cancers were classified as smoking‐ or alcohol‐related as listed by the International Agency for Research on Cancer (International Agency for Research on Cancer, 2019) and double‐checked by the medical oncologist in the PSY‐CA steering group (AdG; see Table 2). Cancer site is determined with codes from the International Classification of Diseases (ICD) version 10 or ICD‐Oncology version 3 codes for the majority of cohorts, with few based on ICD version 9 codes. Only the first cancer diagnosis during follow‐up is considered, and (where available) in situ carcinomas and neoplasms of undetermined behavior are included as the latter could be malignant and the former may develop into cancer later if left untreated. Analyses are also done excluding those with carcinoma in situ and cancers with undetermined behavior to explore whether conclusions hold. All studies determine cancer diagnoses through linkage with national cancer registries, with the exception of CARTaGENE and Rotterdam Study. In both these cohorts, other registries or databases are used to supplement missing information from cancer registries including hospital visits, insurance claims, and GP records. The date of cancer diagnosis is considered as the date of cancer incidence. Where cohorts only provided month and year, a fixed date (15th) is applied for day of diagnosis of cancer. Where cohorts only provided year of diagnosis, June 30th is assumed to be the date of diagnosis.

**TABLE 2:** Overview cancer sites considered smoking‐related and/or alcohol‐related (ICD10 codes)

Both smoking‐ and alcohol‐ related cancer sites	Smoking‐related cancer sites	Alcohol‐related cancer sites
Tongue (C01)	Nasopharynx (C11)	Liver and intrahepatic bile ducts (C22)
Other and unspecified parts of the tongue (C02)	Stomach (C16)	Breast (C50)
Gum (C03)	Liver and intrahepatic bile ducts (C22)	
Floor of mouth (C04)	Pancreas (C25)	
Palate (C05)	Nasal cavity (C30, excluding C30.1—middle ear)	
Other and unspecified parts of mouth (C06)	Accessory sinuses (C31)	
Tonsil (C09)	Bronchus and lung (C34)	
Oropharynx (C10)	Cervix uteri (C53)	
Piriform sinus (C12)	Ovary (C56)	
Hypopharynx (C13)	Kidney (C64)	
Other and ill‐defined sites in lip, oral cavity and pharynx (C14)	Renal pelvis (C65)	
Oesophagus (C15)	Ureter (C66)	
Colorectal (C19‐C20)	Bladder (C67)	
Larynx (C32)	Myeloid leukemia (C92)	

### 2.8 Harmonization and data cleaning

Data harmonization ensures the quality of the results, and the interpretability. Harmonization of data across cohorts also enables the use of standardized scripts for stage 1 analyses (see statistical analysis below) requiring minimal user input. We apply the Maelstrom guidelines (Fortier et al., 2016) to create individual harmonization manuals for each cohort providing guidance on how to recode data to create the variables required for PSY‐CA. Definitions of the variables to be derived are agreed upon within the consortium. Local researchers at each cohort harmonize the data and receive a script to run a number of basic checks (e.g., checking for proportion of missing data). The basic checks are then reviewed by two researchers (LvT and MB) as an additional check of adherence to the manuals.

#### 2.8.1 Missing data

Previously defined, cohort‐specific approaches to dealing with missing data is applied within a given measure (e.g., questionnaire). Where no such approach has previously been defined, the general rule applied is to substitute person‐mean for up to 20% missing responses for a measure. This rule is based on previous studies comparing ways to deal with item‐level missing data, specifically in measures of depression (Bono et al., 2007; Shrive et al., 2006). Missing responses or responses equivalent to “I don't know” are coded as missing data. The only exception is the family history of cancer variables where “I don't know” is coded as no family history of (the specific) cancer.

#### 2.8.2 Unlikely values and extreme outliers

Local researchers harmonizing the cohort data are instructed to investigate extreme, unlikely values and recode these to missing if there is sufficient support that these are errors (e.g., a very high BMI that is markedly higher than the BMI reported at a follow‐up wave for a given participant).

Across all cohorts, extreme outliers are defined as values that are more than three times the interquartile range above the third quartile or below the first quartile, and are truncated to the cut‐offs, respectively. The exception to this rule is variables that contain true zeroes as the lowest possible score as these are likely to be skewed (e.g., pack years where all never smokers score zero). For these variables, only the upper extreme values are truncated. The number of cases that are capped, and the replacement value are recorded for all cohorts and double‐checked.

### 2.9 Statistical analysis

PSY‐CA employs a two‐stage design. In stage 1, local researchers at the cohort level run a standardized R script over the harmonized dataset, and subsequently provide all output generated to the main researchers (LvT, MB, and K‐YP). In stage 2, the output from stage 1 across all cohorts is pooled in the meta‐analyses. As such, the main researchers do not have direct access to cohort raw data. However, in the event that further clarification is required from specific cohort data, subsequent scripts are sent to the local researchers to gain additional information.

#### 2.9.1 Stage 1

For the analyses related to question one (relationship between psychosocial factors and cancer) and question two (interaction), Cox regression models are used. For question three (mediation), different regression models (logistic and multiple) are used to test the path between the psychosocial factor and the mediator, dependent on whether the mediator is categorical or continuous. Cox regression models are used for the path between the psychosocial factor and cancer, and between the mediator and cancer.

Across all research questions, entry age is the age at baseline, while exit age is the age at cancer incidence, death, or end of cancer follow‐up period of the respective cohort (whichever comes first). Note that several cohorts are ongoing but, for the purposes of PSY‐CA, are capped to the moment of linkage with the cancer or vitality registry (whichever comes first). Where another type of cancer occurs (e.g., lung cancer) after cancer endpoint being modeled (e.g., breast cancer), participants are censored at the age of first diagnosis (Ji et al., 2020).

For the first two research questions, the following models are run:

Model 1: univariable—which includes the year of birth and the psychosocial factor.
Model 2: minimum—which additionally adjust for: education (high, low, with mid‐level as reference category) and country of birth (i.e., whether the participant and [where information available] his/her parents were born in the country where the cohort is measured). These confounders are available in all studies with the exception of education in one subcohort of the HUNT cohort where occupation is used instead (high‐ranking profession, low‐ranking profession, with mid‐level as a reference category). Furthermore, in this model we stratify the baseline rates on sex (with the exception of three all‐female studies, and sex‐specific cancer endpoints).
Model 3: maximum—which additionally adjust for, where available, the following measured at baseline:

- All cancer outcomes: current anti‐depressant use, weekly alcohol intake, hours of physical activity per week (metabolic equivalent [MET], if available), body mass index (BMI), pack years, and smoking status (former, current, with never smokers as reference), family history of cancer (where information about family history of cancer endpoint are not available, e.g., family history of breast cancer where breast cancer is the endpoint).
- Breast cancer outcome: parity (distinguishing between three or more [full‐term] pregnancies, one to two pregnancies, with zero pregnancies as the reference category), contraceptive use, menopause status, age at menarche, and a family history of breast cancer.
- Colorectal cancer outcome: sedentary behavior and family history of colorectal cancer.
- Lung cancer outcome: a family history of lung cancer.
- Prostate cancer outcome: a family history of prostate cancer.

Covariates were included where there was no more than 40% missing. A number of the variables adjusted for are considered to potentially interact with psychosocial factors for the second research question, and as mediators for the third research question. Across all models, subgroup analyses are conducted by sex. Furthermore, maximum models are explored where only covariates with no more than 10% missing are included, and excluding education (which may overcorrect for the role of health behaviours), and the minimum model is rerun with the sample of the maximum model for comparability. All additional models, subgroup and sensitivity analyses (outlined below in specific research questions) are considered explorative.

#### 2.9.2 Specifics of research question one

Additional subgroup analyses are conducted based on: cancer stage at diagnosis (stages 1–2, stages 3–4), age group (younger [18–40], mid [41–64], and older [65+]). Additional sensitivity analysis is conducted where borderline or underdetermined cancers and carcinoma in situ are not considered cancer diagnosis (i.e., not an event), and these participants are censored at the moment of this diagnosis. Further sensitivity analysis is conducted where follow‐up is capped to 5 years, 10 years, 15 years, and 20 years (where possible). When testing the role of the psychosocial factors other than depression, an additional model includes symptoms of depression or depression diagnosis (if available in the cohort) along with all the confounders listed above to explore the specificity of the psychosocial factor in the risk of cancer. Furthermore, additional explorative analyses include all psychosocial factors entered simultaneously in the model.

#### 2.9.3 Specifics of research question two

Cancer risk factors tested are based on smoking, alcohol use, weight, physical activity, sedentary behavior, sleep duration and sleep quality, age, sex, education, hormone replacement therapy, and menopausal status. Interaction is assessed by first entering the main effects of the psychosocial factor, the cancer risk factor and an interaction term between the psychosocial factor and cancer risk factor into the Cox models outlined above, to test for multiplicative interaction. Effect estimates of the psychosocial factor, cancer risk factor, and the interaction term are then used to calculate the relative excess risk due to interaction (RERI), a measure of additive interaction. Where interactions are significant, interpretation of the interaction effects is derived as follows: four categories are created (2 [high/low psychosocial factor] ×2 [high/low cancer risk factor]), and included in the Cox models outlined above. Further interpretation is derived by estimating the effect of psychosocial factors within subgroups of the cancer risk factor (high vs. low cancer risk factor) and estimating the effect of the cancer risk factor within subgroups based on psychosocial factor (high vs. low psychosocial factor). Additional subgroup analyses may be explored depending on the results from research question one.

#### 2.9.4 Specifics of research question three

Mediators tested are smoking, alcohol use, weight, physical activity, sedentary behavior, sleep duration, and sleep quality. Mediators are measured at baseline. Three paths are tested in the mediation analyses (for each mediator): path a (psychosocial factor to mediator), path b (mediator to cancer incidence), and path c (the direct path from psychosocial factor to cancer incidence while controlling the mediator). Path a is estimated with linear regression (for continuous mediators) and (multinomial) logistic regression (for categorical mediators). Cox regression models are used to estimate paths b and c. The indirect effect is the product of a × b, the direct effect is c’, and the total effect is a × b + c. Additional explorative analyses will be conducted where all mediators are entered simultaneously in the models. Subgroup analysis by sex is conducted. Additional subgroup analysis will explore differences in cancer stage at diagnosis (stages 1–2, stages 3–4) specifically when looking at physical activity as a mediator in prostate cancer. Further explorative analyses may be performed based on the findings from research questions one and two.

#### 2.9.5 Stage 2 (meta‐analysis)

Random‐effects meta‐analyses are performed. Cohorts are included in a given meta‐analysis when there are at least 10 cancer events and the sample size of the cohort (subgroup) is at least 200. Leave‐one‐out analyses are conducted to identify influential cohorts. Cohorts are considered influential if, upon exclusion of the cohort, the between‐study heteogeneity or effect size substantially changes.

Specifically, for question one, the hazards effects (and robust standard errors) of the psychosocial factors are meta‐analyzed. For question two, meta‐analyses are conducted on hazard effects of the psychosocial factor × cancer risk factor interaction terms (multiplicative interaction) and on the RERI estimates (additive interaction). In question three, overall estimates of all the paths and indirect effect are obtained by carrying out separate univariate meta‐analyses with random effects.

Sensitivity analyses will be performed (where at least two studies with sufficient power are included in the meta‐analysis) for cohorts that determine depression and anxiety through use of clinical interviews. Additional sensitivity analyses are conducted including only cohorts that are recruited from the general population. Finally, moderators of effect size are explored including when the cohort started, and whether the cohort took place in the Netherlands or not.

### 2.10 Power analysis

To test the power of our IPD meta‐analysis, we ran a power simulation study similar to that of Ensor et al. (2018), with a focus on depression. The study information that we used for the simulation study involved the total number of participants, the prevalence of depression at the baseline measurement of the study (estimated where this was not yet known), and the expected number of cancer cases of two types of cancer: lung cancer and any cancer (anticipated smallest and largest categories, respectively). Requiring 80% power, an alpha of 0.05 (two‐sided testing), and using fixed‐effects meta‐analysis of HR from Cox regression models, our simulation study showed that regarding main effects the minimal detectable effect size for is HR = 1.04 for any cancer and HR = 1.12 for lung cancer.

Regarding the interaction analyses, our calculations (based on the general convention that the sample size in a single trial should be increased approximately four times to detect the interaction effect (Brookes et al., 2004; McClelland & Judd, 1993)) showed that the minimal detectable effect size for any cancer is HR = 1.08 for any cancer and HR = 1.25 for lung cancer. Regarding the mediation analyses, our calculations (based on an inflation factor of two, compared to testing the main effects) showed that the corresponding minimal detectable effect size is HR = 1.06 for any cancer and HR = 1.18 for lung cancer. The inflation factor of two for mediation analysis was found as an upper bound by comparing sample sizes needed for main effects with those for mediation effects using Baron and Kenny's test assuming different effect sizes (Fritz & MacKinnon, 2007).

### 2.11 Interpretation

The hypotheses tested include four psychosocial factors (depression [symptoms], anxiety [symptoms], recent loss events, and perceived social support), four health behaviors (smoking, alcohol, physical activity, and weight), and seven cancer outcomes (see Appendix 1). It is important to specify that the interpretation of the results is done holistically, and not based on a single association (i.e., “cherry picking”). Through triangulation of the evidence from the different analyses, we conclude if there is statistical support of an association between psychosocial factors and cancer, and by extension whether there is evidence for interaction of or mediation by health behaviors. Interpretation is done by looking at the obtained HRs (or beta‐coefficients) and 95% confidence intervals (CIs) and by exploring consistency and robustness of the findings. Additionally, the associations between a number of further psychosocial factors, other health and demographic factors, and cancer are studied. The results of these additional analyses are considered to be exploratory. Subgroup and sensitivity analyses are considered to be exploratory as well.

## 3 DISCUSSION

Previous meta‐analyses investigating the role of psychosocial factors in cancer incidence have shown mixed findings (Chida et al., 2008; McGee et al., 1994; Oerlemans et al., 2007). While this is partly explained by differences in the types of psychosocial factors and cancer endpoints, many studies in these meta‐analyses pose further limitations, in particular the absence of adjustment for key confounders. PSY‐CA aims to elucidate the relationship between psychosocial factors and cancer incidence by employing clearly defined psychosocial factors measured with reliable instruments. Through harmonization of the data across cohorts, strict definitions of the psychosocial factors are applied, thereby increasing interpretability. Furthermore, PSY‐CA considers several different cancer endpoints in addition to any cancer endpoint, which is crucial given the distinct etiologies of cancers. By employing two‐stage IPD meta‐analyses, PSY‐CA can address limitations of previous traditional meta‐analyses such as adjusting for key confounders in all cohorts. Therefore, the results from PSY‐CA are more reliable and interpretable.

Given the link of health behaviors such as smoking with both psychosocial factors (Strine et al., 2008; Verger et al., 2009) and cancer (Biswas et al., 2015; Chen et al., 2018; Kerr et al., 2017), there is a need to clarify whether the role of health behaviors is more than a confounding effect. Health behaviors, demographic and somatic factors that are well established cancer risk factors may interact with psychosocial factors to pose further risk. Furthermore, health behaviors could explain the link between psychosocial factors and cancer (i.e., health behaviors as mediators). Research into the role of health behaviors in the association between psychosocial factors and cancer is surprisingly lacking, and PSY‐CA aims to provide insight into this area. As such, the results from the proposed study outlined in this article may reveal psychosocial factors that put individuals at risk for cancer, identify certain subgroups to target with preventive interventions, and support the use of health‐behavior interventions to reduce the risk of cancer associated with psychosocial factors.

## CONFLICT OF INTEREST

The authors declare no conflict of interests.

### PEER REVIEW

The peer review history for this article is available at https://publons.com/publon/10.1002/brb3.2340

## APPENDIX 1

### Specific hypotheses by research questions

Research question 1: Do psychosocial factors (depression, anxiety, perceived lack of social support, relationship status, recent loss events, neuroticism, and general distress) increase the incidence of cancer? Specifically, we hypothesize that depression, anxiety, recent loss events, and perceived lack of social support all individually increase the incidence of any cancer, breast cancer, lung cancer, prostate cancer, colorectal cancer, smoking‐related cancers, and alcohol‐related cancers. We limit our hypotheses to depression, anxiety, recent loss events and perceived low social support given the rather clear distinction between these concepts (e.g., while neuroticism and general distress are relatively broad constructs incorporating symptoms of both depression and anxiety), and the focus on these factors in prior research (e.g., relatively little research has looked at relationship status and cancer incidence). Therefore, analyses relating to neuroticism, general distress and relationship status are considered explorative.

Research question 2: Do these psychosocial factors interact with health behaviors (smoking, alcohol use, weight, physical activity, sedentary behavior, sleep duration, and sleep quality) or demographic and clinical factors (age, sex, education, hormone replacement therapy, and menopausal status) on the risk of cancer incidence? Specifically, we hypothesize that the risk of cancer in people with psychosocial stress (i.e., elevated depression symptom level or diagnosis, elevated anxiety symptom level or diagnosis, a recent loss event, or perceived lack of social support) and unhealthy behavior (smoking, alcohol use, overweight, and low physical activity) is greater than the sum of the individual effects of the psychosocial factor and unhealthy behavior on cancer incidence. We limit our hypotheses to these health‐related behaviors given the consistent evidence of their association with cancer.

Research question 3: Are the relationships between these psychosocial factors and incidence of cancer mediated by health‐related factors (smoking, alcohol use, weight, physical activity, sedentary behavior, sleep duration, and sleep quality)? Again limiting the hypotheses to depression, anxiety, recent loss events, and perceived low social support, we hypothesize that (a) smoking, alcohol use, physical inactivity, and high body mass index (BMI) partially mediate the association between the psychosocial factors and cancer of any kind, breast cancer, and colorectal cancer; (b) smoking partially mediates the association between the psychosocial factors and smoking‐related cancers; (c) alcohol use partially mediates the association between the psychosocial factors and alcohol‐related cancers; (d) smoking and physical inactivity partially mediate the association between the psychosocial factors and lung cancer; and (e) physical inactivity partially mediates the association between the psychosocial factors and prostate cancer.
